# Reproduction success in European badgers, red foxes and raccoon dogs in relation to sett cohabitation

**DOI:** 10.1371/journal.pone.0237642

**Published:** 2020-08-14

**Authors:** Krzysztof Nowakowski, Agnieszka Ważna, Przemysław Kurek, Jan Cichocki, Grzegorz Gabryś

**Affiliations:** 1 Department of Zoology, University of Zielona Góra, Zielona Góra, Poland; 2 Department of Plant Ecology and Environmental Protection, Adam Mickiewicz University, Poznań, Poland; University of Jyväskylä, FINLAND

## Abstract

The setts of the European badger *Meles meles* can be cohabited during reproductive season by the red fox *Vulpes vulpes* and raccoon dog *Nyctereutes procyonoides*. There is no information on the possible impact of both species on the size of badgers’ litter. The aim of the study was to show the influence of cohabitation of the same setts by badger, raccoon dog and fox on the litter size. The research was conducted in 2012–2014 and 2018 in the lowland forests of western Poland. We conducted the survey of setts by direct observations and analysis of photographic material from trap cameras during mid-April–July each year. We recorded 85 badger litters, 18 fox litters, and 15 raccoon dog litters. Average litter size was 1.71 (±0.90), 2.44 (±1.34) and 4.93 (±2.76) litter mates in badgers, foxes and raccoon dogs, respectively for all observed pairs. Badger litter size did not differ between setts used only by badgers including pairs with no cubs (1.66 ± 0.98) and cohabited with foxes (1.90 ± 0.32) or raccoon dogs (1.88 ± 0.81). However, foxes reared even more cubs in setts cohabited with badgers than when badger was absent (2.90 ± 1.37 vs. 1.88 ± 1.13 respectively). In the case of raccoon dogs, there were no differences in the mean number of their cubs in setts with badgers (5.25 ± 2.92) and without badgers (4.57 ± 2.76). The results indicate that the cohabitation of setts by badgers, foxes and raccoon dogs does not affect litter size negatively.

## Introduction

Physical engineered structures such as dug burrows can provide shelter for other terrestrial vertebrates and positively affect their breeding success [1]. Burrows provide microhabitats that increase species richness and abundance [2, 3]. Thus, many burrowing mammals are considered to be crucial in ecosystem functioning [4]. Although the knowledge about the interspecific interactions between mesocarnivores cohabiting the same burrows is accumulating, the data is scarce and further investigations are needed [e.g. 5–9]. There is an increasing evidence of mammals cohabitation of burrows. The effects of cohabitation on the breeding are unknown. Here we assess cohabitation of three mesocarnivores and the effects of cohabitation on the litter size.

European badgers *Meles meles* occupy several setts that vary in size and function. Some serve as temporary shelters and some are used during the breeding season. Setts used in the breeding season are composed of underground corridors, chambers and dozens of tunnels and entrances [10]. These setts are also used as winter dens [10, 11]. The setts are used for several years and may reach considerable sizes. Setts’ microclimate guarantees convenient shelter during severe weather in winter and summer [12, 13]. Badgers’ reproductive setts are the largest in terms of volume in comparison with burrows of other European mesocarnivores, e.g., red fox *Vulpes vulpes*. The badger family group usually uses only some of the setts, which makes the other look abandoned. Nevertheless, the extensive area of the setts may reduce competition between the cohabitants [14] and limits the spread of ectoparasites [15].

Main setts can be used even for several decades by many generations of badgers, making them permanent and crucial elements of the local ecosystem [16–18]. Thus, badgers’ setts act as hotspots for plants [19] invertebrates [20], amphibians [21], reptiles [22], and mammals [7, 21, 23–27]. Many authors reported badgers dwelling setts with red foxes [7, 8, 21, 28, 29] and raccoon dogs *Nyctereutes procyonoides* [28, 30–32] or even all three species together [29]. Those mesocarnivores may cohabit more than 50% of badger setts [5].

In central Europe until the second half of the 20th century, mainly red foxes cohabited badgers setts or used the abandoned burrows of this predator. Red fox is a common native predator digging short breeding dens with few entrances [33]. Dens are located mainly on the edge of forest [34] or in opened areas as fields and meadows because the red fox, as an environmental opportunist, can inhabit various habitats, as woodlands, agricultural [35] and urban areas [36].

Raccoon dog appeared in the central part of the continent after the introduction in the European part of Russia. Over the decades, it has gained the status of a common species with an increasing population and inhabited area [37]. In Poland, raccoon dogs were observed in the mid–20th century and by the end of the century they inhabited almost entire territory of the state [37]. The racoon dog is an opportunist, whose existence is limited mainly by the availability of food [38]. In the primeval forests, raccoon dogs find shelter in uproots, under fallen trees and in other natural hiding places [39].

Observations of raccoon dogs in badgers’ setts are becoming more common throughout the European range of the species. Badgers’ setts are inhabited by raccoon dogs as temporary shelters and places for rearing the cubs and wintering [29, 30]. The frequency of sett colonization by raccoon dogs depends on the density of the latter species and when the density of raccoon dogs increases, the frequency of colonisation of badgers’ setts grows [32]. Some authors even claim that the raccoon dogs’ success in invading Europe is due to the possibility of surviving unfavourable winters in badgers’ setts where they find good shelter from frost and predators [39].

It is difficult to conclude unequivocally whether the burrows are accidental or long–term refuges or breeding places for the cohabiting species. In many cases there was no straight evidence of cohabitation of two or more carnivores in the burrows and the authors of studies deduced it basing on certain signs (i.e. footprints) in the vicinity of badger sett entrances. It is important to determine what kind of interaction between hosts and guests are observed–are they only episodic (only visits) or maybe more complex, such as breeding in the same sett. Moreover, it is not known how cohabitation of the same setts by different species of medium–sized carnivores affect reproduction of the hosts and guests.

The aim of this study was to find how cohabitation of the same setts may affect reproduction characteristics in medium–sized carnivores. Basing on information presented above, we put forward two hypotheses: 1) Carnivores minimize antagonistic interactions by avoiding the setts inhabited by the host, the badger, and 2) The number of cubs of badgers, foxes, and raccoon dogs emerging from the setts is lower in badgers’ setts inhabited jointly than in setts inhabited separately by each species.

## Material and methods

### Study area

The research was carried out in a lowland region of western Poland near Trzciel. The study area covered 389.5 km^2^ (52°17'–52°32' N, 15°30'–16°01' E) with a predomination of forest and field mosaic in landscape. Forests, grouped into 213 complexes, the area of which varied from one to more than 2000 hectares, occupy 52% of the research area. Scots pine *Pinus sylvestris* on sandy soils is a predominant species. Four small rivers flow through the research area. The total area of lakes in the studied area is about 1350 ha. The mild climate prevails with the average annual temperature 10.5°C. The coldest month is February with mean temperature: –4.1°C, the warmest is August with mean: 21.7°C [40]. The average human population density in this region is 42 people per km^2^ [41].

### Data collection

In the years 2012–2014 and in 2018 we monitored 94 badger setts to count the number of badger adults and young. 30 of the monitored setts were used as breeding sites by badgers. Analogous data were collected in the same setts in relation to the species that cohabited badgers’ setts–foxes and raccoon dogs. We observed badgers’ main setts where cubs were reared or at least those which were occupied by adults. The studies were conducted from mid–April to the end of July when badgers were the most active outside the setts [42]. Mean number of available entrances in monitored setts was 6 (range: 3–17). Mean number of used entrances was 3 (range: 0–10).

Data on the species assemblage and abundance of individuals inhabiting setts were collected using two corresponding methods, direct observations and video recording. The direct observations at the setts were conducted in the evening (5PM–10PM) from portable platforms located 30–40 meters from the setts. We recorded the total number of individuals, adults and juveniles, separately. We repeated the observations at the setts two or three times during the season until the number of family members was determined. The data obtained during the observations were supplemented with information from camera traps. These were carried out from mid–April to July. We used a total of 12 camera traps (models Ecotone HE–30, SGN– 5220 and Maginon 90258). To be more accurate in estimation of animals (adults and cubs) dwelling in the burrow, in some cases we used even six camera traps at the same sett placed near (3–10 meters) the most intensively used entrances. The camera traps registered all the moving animals (badgers and other mammals) for two to seven days. During the study we conducted 758 observations of badger dens (Table 1).

**Table 1:** The details of European badgers’ setts monitoring in 2012–2014 and in 2018 in western Poland.

Year of study	Direct observation			Camera traps			First cubs observation
	No. of nights	No. of setts	Observation period	No. of nights	No. of setts	Observation period	
**2012**	60	20	1 V–30 VI	57	18	26 V–15 VII	2 V
**2013**	50	25	1 V–30 VI	108	36	1 V–8 VII	3 V
**2014**	50	25	1 V–30 VI	205	38	14 IV–30 VI	23 IV
**2018**	50	25	1 V–30 VI	178	33	1 VI–30 VI	2 V
**Cumulative total**	**210**	**95**	**–**	**548**	**125**	**–**	**–**

We distinguished two kinds of cohabitation of carnivores in the setts. First type occurred when the presence of adult individual or individuals with no signs of breeding was recorded (accidental visits, using as short time shelter, etc.). The first type of cohabitation was recorded more frequently than the second type, which was when the presence of cubs of two species in the same sett was observed. Therefore we focused on setts with litters because we decided that cohabitation of setts really existed when cubs of both carnivores were present. We excluded from further analysis setts functioning as accidental places of refuge where the studied carnivores were recorded only once or where were no signs of breeding such as presence of both sexes, food delivering, or traces of lactation. Also records of carnivores in the neighborhood but not entering the setts, were not analysed. Such approach excluded accidental visits of non–breeding individuals that could not be considered in term of sett cohabitation.

The computations referring to the mean number of cubs were conducted for data obtained when cubs emerged from setts and became almost independent. The analyses also included setts where no young badgers, foxes or raccoon dogs were found, but only if two adults with signs of reproduction (nursing female, cubs) were recorded. This approach included all pairs (also with no breeding success when no cubs emerged from the sett) because the presence of another species of carnivore in the sett could be one of the reasons why the young were absent.

### Statistical analysis

The number of breeding pairs of each carnivore varied between years as a result of natural fluctuations. As a consequence unequal number of samples from each year of investigation were obtained and thus we used factorial ANOVA for unbalanced designs (for Type–III sums of squares) to estimate the significance in differences between the number of cubs including year and status of the brood (sett cohabited with other carnivore or not) as factors. In the case when interaction year × status was insignificant we excluded it from the analysis and tested only factors. Prior to analyses, the skew data were transformed with logarithmic or exponential functions to obtain a normal or at least symmetric distribution. Binomial logistic regression with glmer function was applied to assess the relations between the number of adult badgers inhabiting the sett (fixed variable) and the presence (1) or absence (0) of fox and raccoon dog in the same sett. Variables: year and sett were considered as random effects. All analysis were performed with Rv3.5.3 [43] using the ‘lme4’ [44], ‘ggplot2’ [45], ‘ggthemes’ [46] and ‘car’ [47] packages.

### Permits

We have conducted the research on game species not strictly protected by the Polish law. Direct observations and video recording of mammals that are not on the list of strictly protected species do not require permissions of institutions for protection of nature. Our research didn't require catching or any other activities that induce stress and didn't require the permit from the Local Ethical Committee on Animal Testing (ECAT is a committee at the Polish Ministry of Science and Higher Education).

## Results

### Setts cohabitation and number of cubs

**Fig 1:**
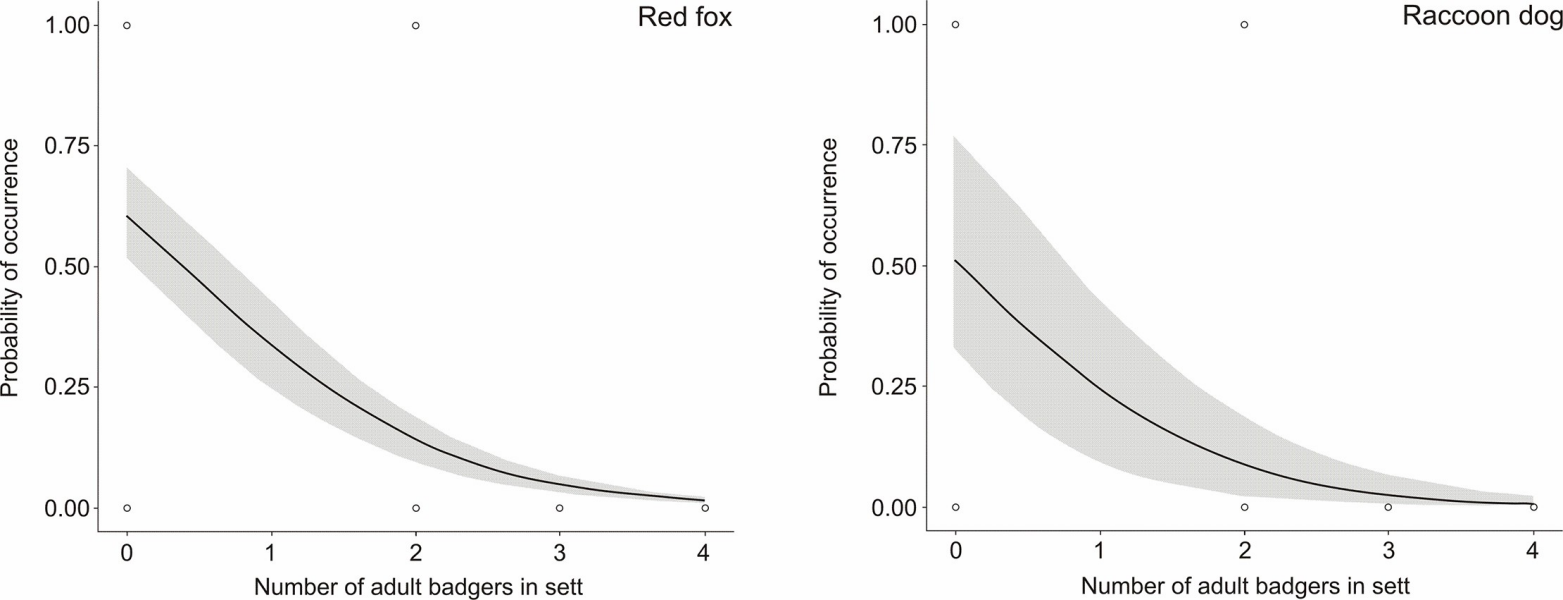
Red fox and raccoon dog probability of occurrence (95% confidence intervals) in relation to the number of adult European badgers observed in the setts in 2012–2014 and in 2018 in western Poland.

In the years 2012–2014 and in 2018, from mid–April to July, we recorded data from 85 badgers, 18 foxes and 15 raccoon dogs breeding pairs (S1 Appendix). In total, during the study period we recorded 100 broods of three species of carnivores in badger setts–badgers, foxes and raccoon dogs. In 33 badger setts (abandoned and/or used by the hosts–badger) broods of foxes and raccoon dogs have been recorded. 56% of foxes (N = 18) and 53% of raccoon dogs (N = 15) breeding pairs reared their cubs when badgers with their cubs were present in the same sett. We recorded badger–fox mean inter–annual cohabitation of setts (cubs of both carnivores present in the sett) in 9.8% of records and mean badger–raccoon dog cohabitation in 7.6% of records (Table 2). There were no cases when all three analyzed carnivores cohabited the same sett in our research. We found that fox (*Z* = -2.804, *P* = 0.005) or raccoon dog (*Z* = -2.111, *P* = 0.035) do not utilize setts when the number of adult badgers exceeded two individuals in the sett (Fig 1).

**Table 2:** European badger, red fox and raccoon dog cohabitation of setts with breeding pairs in western Poland (only data concerning cohabited setts presented, total number of setts inhabited only by red fox or raccoon dog was higher).

Species	Year							
	2012 (N = 23)		2013 (N = 27)		2014 (N = 21)		2018 (N = 29)	
	n	%	n	%	n	%	n	%
**Badger–fox**	4	17.4	0	0.0	1	4.8	5	17.2
**Badger–raccoon dog**	3	13.0	0	0.0	0	0.0	5	17.2

N–total broods of all species, n–number of cohabited broods.

Mean number (±SD) of badger cubs including also setts cohabited with other carnivores ranged with 1–5 cubs and (mean: 1.71 ± 0.90) for all pairs (N = 85), and 1.88 (± 0.74) for pairs excluding those with no litter (N = 77). Mean number of red fox cubs for all pairs (N = 18) was 2.44 ± 1.34 (range: 1–6 cubs) and for raccoon dogs (N = 15) 4.93 ± 2.76 (range: 0–12 cubs). The mean number of cubs for all carnivores did not differ significantly between years (Table 3). During the study we recorded only five deaths of badgers at setts–two young and three adults. No cases of death of red foxes and raccoon dogs were recorded.

**Table 3:** Mean number (SD) of cubs for three carnivores inhabiting European badgers’ setts observed in western Poland.

Species	Year								*F*	*P*
	2012	N	2013	N	2014	N	2018	N		
**European badger (all pairs)**	1.45 (0.89)	20	1.91 (1.06)	22	1.80 (0.83)	20	1.65 (0.78)	23	0.027	0.869
**European badger (pairs with cubs)**	1.93 (0.26)	15	2.00 (1.00)	21	1.89 (0.74)	19	1.73 (0.70)	22	2.311	0.133
**Red fox (all pairs)**	2.00 (0.89)	6	1.33 (0.58)	3	–^1^	1	2.75 (1.04)	8	1.760	0.204
**Raccoon dog (all pairs)**	3.00 (2.45)	4	6.50 (3.54)	2	–^1^	1	5.50 (2.78)	8	1.240	0.287

Data pooled for all setts (including setts inhabited by both species). ^1^ –in 2014 there was only one litter recorded in red foxes (six cubs) and one litter in raccoon dogs (five cubs). N–number of pairs.

### Badger cubs and red fox or raccoon dog presence in the sett

The mean number (±SD) of badger cubs (including pairs with no litter) did not differ significantly between setts cohabited with foxes (1.90 ± 0.32), raccoon dogs (1.88 ± 0.64) and setts occupied only by badgers (1.66 ± 0.98) (*F*_2, 81_ = 0.592, *P* = 0.556). Also the mean number of cubs for badger pairs excluding those with no litter did not differ significantly between setts cohabited with foxes (1.90 ± 0.32) and raccoon dogs (1.88 ± 0.64) and setts occupied only by badgers (1.88 ± 0.81) (*F*_2, 73_ = 0.317, *P* = 0.729).

### Red fox and raccoon dog cubs and badger presence in the sett

**Fig 2:**
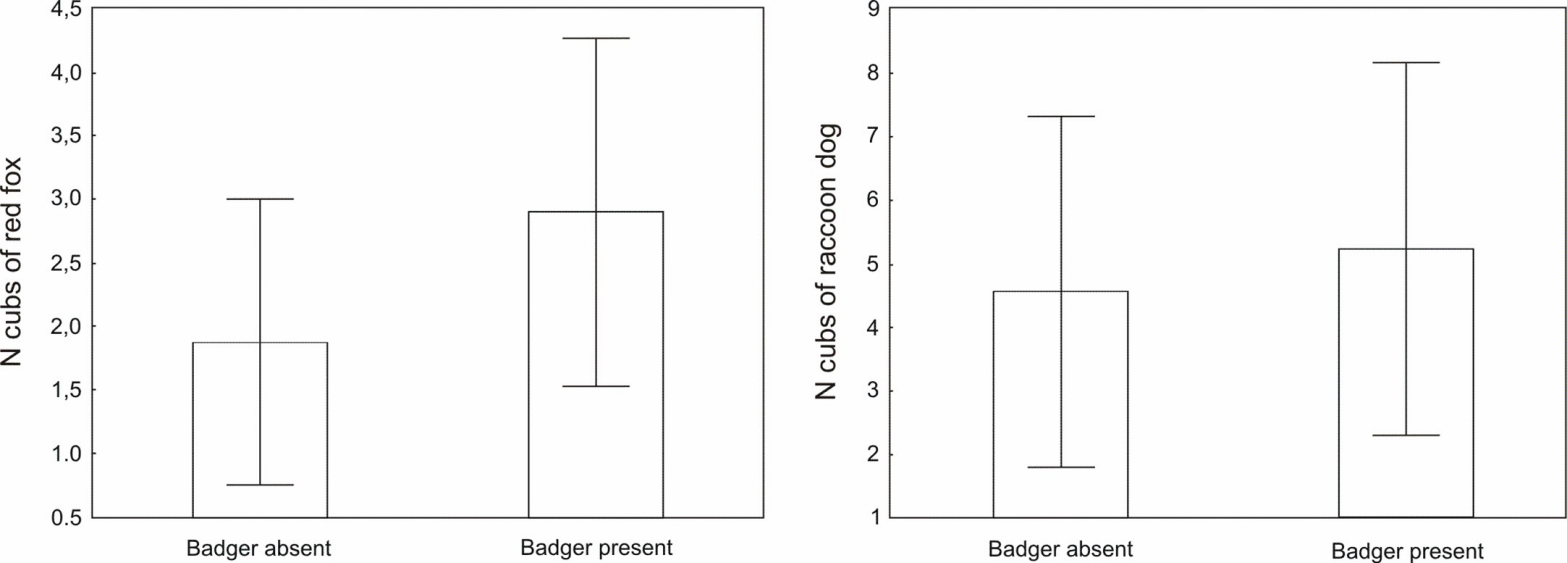
Mean number (±SD) of red fox and raccoon dog cubs in relation to presence/absence of European badger in the sett in 2012–2014 and in 2018 in western Poland.

As in the case of badgers, the mean number of cubs of foxes and raccoon dogs did not differ significantly between years and was highly variable (Table 3). The mean number (±SD) of fox cubs from setts cohabited with adult badgers was surprisingly higher (2.90 ± 1.37) and close to statistical significance (*F*_1, 15_ = 3.359, *P* = 0.087, Fig 2) than when badgers were absent in the sett (1.88 ± 1.13). In the case of raccoon dogs there were no differences (*F*_1, 12_ = 0.170, *P* = 0.687, Fig 2) in the mean number of cubs in setts with (5.25 ± 2.92) and without badgers (4.57 ± 2.76).

## Discussion

This is the first study that describes that cohabitation of burrows by multiple native and alien mesocarnivores does not affect litter size. Our results showed that the cohabitation of the setts by badgers/foxes and badgers/raccoon dogs did not adversely affect the size of badgers’ litter that emerge the setts. In our studies the proportion of setts cohabited by badgers and foxes or raccoon dogs (*sensu* there were cubs of both carnivores in the sett) may reach 17% in one year. For comparison in the Białowieża Forest, studies report 10 records of cohabitation out of 15 surveyed badger setts (five records of raccoon dogs and five records of foxes) [29]. In central Poland, the setts cohabited by badgers and foxes accounted for 13% of surveyed dens, while no raccoon dogs were recorded at all [28]. Also, in the Carpathians (southern Poland) only 7% of badger setts were cohabited with foxes (30 observations) [21]. There are some reports concerning the cohabitation of the same setts by all three species but it happens sporadically– 4% of setts [29]. The simultaneous occupancy of setts and the raising of young badgers and foxes have been reported many times [23, 27, 48, 49]. In the case of raccoon dogs, the information concerning the use of the same setts with badgers also occurs [50].

Breeding success of badgers depends also on intraspecific interactions including density of population or social and class age structure of females [51]. The average number of badger cubs in a litter in western Poland was lower than in other regions of Poland and Europe, including the areas with higher badger population density [52]. In central Poland there were three cubs per litter on average, and litters with even up to six young badgers were observed [53]. In the Białowieża Primeval Forest litters with 2–3 young were most common [54]. Lower numbers of litter were recorded in the mountainous part of the country with a maximum of three cubs [55] (Table 4).

**Table 4:** Mean litter sizes in European badgers, red foxes and raccoon dogs observed at setts in different regions of Europe.

Location	Mean number of cubs	Sources
**European badger**		
**W Poland**	1.7	present study
**Central Poland**	3.0	[53]
**Białowieża Forest, Poland**	2.4	[54]
**Carpathian Mts, Poland**	0.6	[55]
**NE Poland**	2.3	[56]
**Netherlands**	3.3	[23]
**SW England**	2.3	[57]
**S and SW England**	2.4	[58]
**E Germany**	2.4	[49]
**Red fox**		
**W Poland**	2.4	present study
**Belarus**	3.2	[59]
**Central Poland**	3.8	[60]
**NE Poland**	6.0	[56]
**W Switzerland**	3.1–4.6	[61]
**Italy**	2.6	[62]
**Raccoon dog**		
**W Poland**	4.9	present study
**E Poland**	6.2	[63]
**Finland**	8.8	[64]
**NE Poland**	5.8	[56]

**Fig 3:**
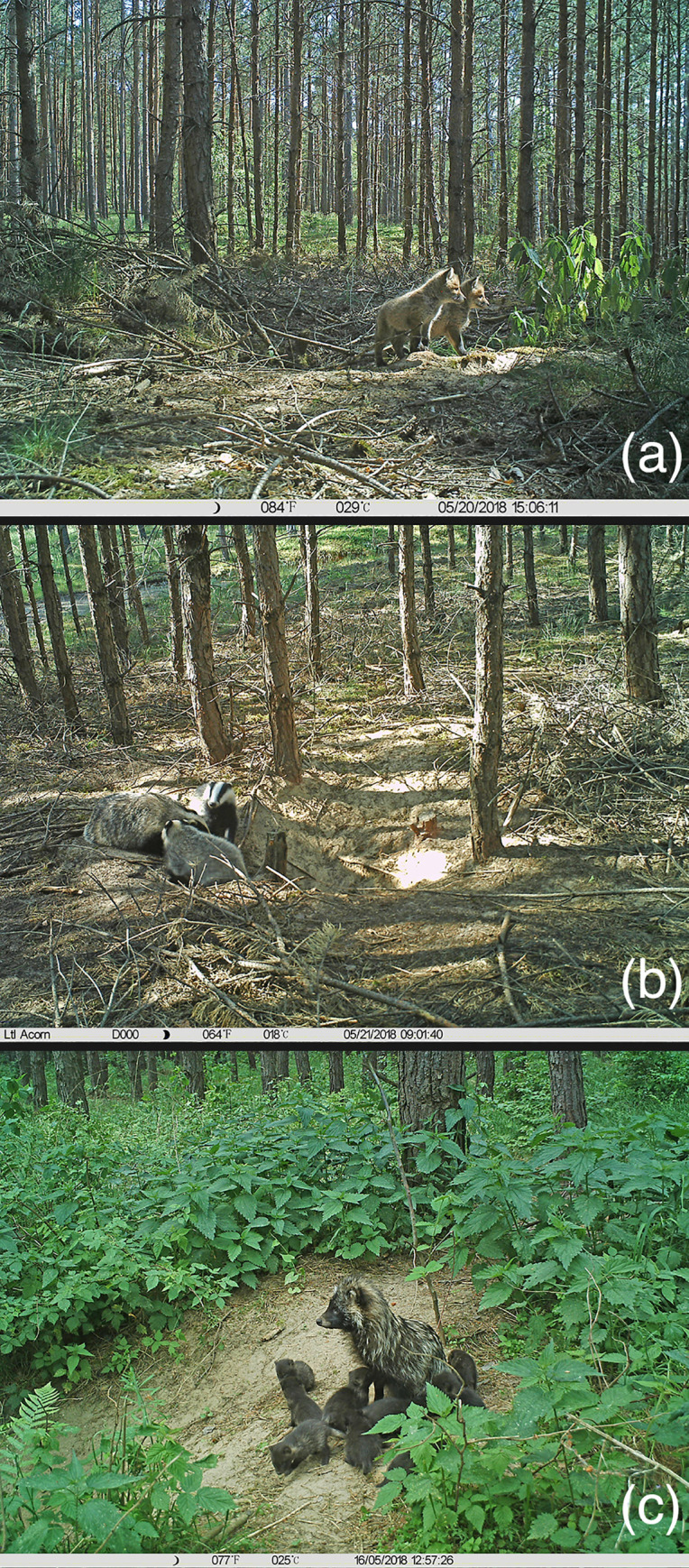
Cubs from cohabited setts in western Poland. (a) (b)–two young red foxes (total) and two young badgers (total) playing with an adult in the same sett, (c)–most numerous litter recorded in our research in raccoon dog (twelve cubs) that cohabited the sett with 2 ad. + 1 juv. of badgers; in the picture, eight out of twelve cubs (May 2018).

Foxes cohabiting badgers’ setts in our study had less numerous litters than in other study areas [56, 59–61, 65]. However, foxes that inhabited the same setts with badgers had higher litter size (close to statistical significance) than in the case of foxes from setts where badgers were absent (hypothesis #2 rejected). It proves at least that the badger presence in the sett does not act as a limiting factor for fox litter size. In our research, raccoon dogs had comparable litter sizes to other European populations [63, 64]. Once, we recorded a litter that consisted of 12 raccoon dog cubs in a sett that was cohabited with adult badgers and their one cub (Fig 3). Unfortunately, there are no published data about foxes and raccoon dogs litter sizes in cohabited badgers setts or in badger setts without the hosts in other European areas.

In cases of shared colonisation of setts by various species of predators (intruders and hosts), antagonistic behaviours are often reported, such as effective removal of the rival from the sett or killing each other’s juveniles [8, 28, 48]. The scale of the threat and the cause of the attacks are not identified. Killing of the cubs happens and should supposedly be interpreted as elimination of a competitive predator [66] rather than predation on cubs. Young badgers, however, may be exposed to raccoon dog attacks when adults leave the sett to search for food [32]. Also, the extensive size of badgers’ setts facilitates spatial separation of the species [29]. In addition, our results showed that raccoon dogs and foxes did not cohabit with badgers when there were more than two adult badgers in the sett. Moreover only half of the red fox and raccoon dog broods were cohabited with badgers. It may suggest the behaviour minimizing antagonistic interactions (hypothesis #1 favored). Aggressive interactions are also observed between adult badgers, nevertheless not often. It is because of complex behavior of avoiding confrontations [67, 68]. The cohabitation of setts with badgers in our studies also did not have a negative effect on the litter size of foxes and raccoon dogs. In Belarus, fatal attacks of foxes and raccoon dogs were recorded on young badgers which, according to the authors, may be one of the causes of the observed decline in the badger population [32]. Fox attacks on young badgers were also reported in Spain [66]. The fur of young badgers was recorded in the faeces of raccoon dogs in the Białowieża Primeval Forest [65]. Cases of killing young badgers are more frequent in smaller family groups [32]. It corresponds with our results showing that red foxes and raccoon dogs avoid setts with badgers families consisting of more than two adults. In Belarus cub killing and aggression were recorded from both sides between badgers and raccoon dogs [32].

There is a lack of data concerning sett cohabitation by medium–sized carnivores (as breeding sites) and reports concerning interaction between them are usually scarce, mostly highlighting aggressive relations, such as killing the cubs. Adult foxes are only chased away by badgers [56]. Their activity patterns suggest also a differentiated use of night-time [69]. Sporadic killing of cubs seems to be confirmed by other authors [39, 48]. It is not without significance that young foxes and raccoon dogs are active during the day and badgers begin their activity before dark. We also observed that young foxes inhabiting badgers’ setts behave extremely carefully and rarely penetrate the burrow area occupied by badgers (Nowakowski K.–unpubl. data). However, our results confirmed that antagonistic interactions between medium–sized carnivores inhabiting the same setts does not influence litter size significantly.

## Supporting information

S1 Appendix Number of breeding pairs of three carnivores inhabiting badgers’ setts. (DOCX)
