# Supplementary material for: Reproduction success in European badgers, red foxes and raccoon dogs in relation to sett cohabitation
Source: PLoS One. 2020 Aug 14;15(8):e0237642. doi: 10.1371/journal.pone.0237642 (PMC7433744; doi:10.1371/journal.pone.0237642)
Supplement: S1 Appendix — (DOCX) [file pone.0237642.s001.docx]

**S1 Appendix Number of breeding pairs of three carnivores inhabiting badgers’ setts.**

| **Species** | **Year of study** | **Setts with one species** | | **Cohabited setts** | |  |
| --- | --- | --- | --- | --- | --- | --- |
|  |  | **Number of cubs** | **Number of pairs** | **Number of cubs** | **Number of pairs** |  |
| **Badger** | 2012 | 15 | 13 | 14 | 7 |  |
|  | 2013 | 42 | 22 | 0 | 0 |  |
|  | 2014 | 34 | 19 | 2 | 1 |  |
|  | 2018 | 20 | 13 | 18 | 10 |  |
|  | **Total** | **111** | **67** | **34** | **18** |  |
| **Fox** | 2012 | 4 | 2 | 8 | 4 |  |
|  | 2013 | 4 | 3 | 0 | 0 |  |
|  | 2014 | 0 | 0 | 6 | 1 |  |
|  | 2018 | 7 | 3 | 15 | 5 |  |
|  | **Total** | **15** | **8** | **29** | **10** |  |
| **Raccoon dog** | 2012 | 0 | 1 | 12 | 3 |  |
|  | 2013 | 13 | 2 | 0 | 0 |  |
|  | 2014 | 5 | 1 | 0 | 0 |  |
|  | 2018 | 14 | 3 | 30 | 5 |  |
|  | **Total** | **32** | **7** | **42** | **8** |  |
